# Hair Cortisone Levels and the Metabolic Syndrome: Stronger Links in Younger Compared to Older Adults

**DOI:** 10.1210/clinem/dgaf322

**Published:** 2025-06-03

**Authors:** Susanne Kuckuck, Robin Lengton, Julius März, Nina van Gerwen, Dimitris Rizopoulos, Manon H J Hillegers, Maryam Kavousi, Sjoerd A A van den Berg, Erik J Giltay, Brenda W J H Penninx, Mariëtte R Boon, Elisabeth F C van Rossum

**Affiliations:** Department of Internal Medicine, Erasmus MC, University Medical Center Rotterdam, 3015 GD Rotterdam, The Netherlands; Obesity Center CGG, Department of Internal Medicine, Erasmus MC, University Medical Center Rotterdam, 3015 GD Rotterdam, The Netherlands; Department of Epidemiology, Erasmus MC, University Medical Center Rotterdam, 3015 GD Rotterdam, The Netherlands; Department of Internal Medicine, Erasmus MC, University Medical Center Rotterdam, 3015 GD Rotterdam, The Netherlands; Obesity Center CGG, Department of Internal Medicine, Erasmus MC, University Medical Center Rotterdam, 3015 GD Rotterdam, The Netherlands; Department of Child and Adolescent Psychiatry/Psychology, Erasmus MC, University Medical Center Rotterdam, 3015 GD Rotterdam, The Netherlands; Department of Biostatistics, Erasmus MC, University Medical Center Rotterdam, 3015 GD Rotterdam, The Netherlands; Department of Biostatistics, Erasmus MC, University Medical Center Rotterdam, 3015 GD Rotterdam, The Netherlands; Department of Child and Adolescent Psychiatry/Psychology, Erasmus MC, University Medical Center Rotterdam, 3015 GD Rotterdam, The Netherlands; Department of Epidemiology, Erasmus MC, University Medical Center Rotterdam, 3015 GD Rotterdam, The Netherlands; Department of Clinical Chemistry, Erasmus MC, University Medical Center Rotterdam, 3015 GD Rotterdam, The Netherlands; Department of Psychiatry, Leiden University Medical Center, 2333 ZG Leiden, The Netherlands; Department of Psychiatry, Amsterdam Neuroscience and Amsterdam Public Health, Amsterdam UMC, Vrije Universiteit Amsterdam, 1081 HV Amsterdam, The Netherlands; Department of Internal Medicine, Erasmus MC, University Medical Center Rotterdam, 3015 GD Rotterdam, The Netherlands; Obesity Center CGG, Department of Internal Medicine, Erasmus MC, University Medical Center Rotterdam, 3015 GD Rotterdam, The Netherlands; Department of Internal Medicine, Erasmus MC, University Medical Center Rotterdam, 3015 GD Rotterdam, The Netherlands; Obesity Center CGG, Department of Internal Medicine, Erasmus MC, University Medical Center Rotterdam, 3015 GD Rotterdam, The Netherlands

**Keywords:** hair glucocorticoids, cortisone, HPA-axis, metabolic syndrome, cardiovascular risk factors, age

## Abstract

**Background:**

The striking link between Cushing syndrome, the metabolic syndrome (MetS), and cardiovascular disease suggests that long-term exposure to high glucocorticoid levels catalyzes cardiometabolic deterioration. However, the relation of subtle variations in long-term glucocorticoid levels with MetS remains poorly understood. Specifically, little is known about potential moderating roles of age, sex, and mental health status in this association.

**Design:**

We investigated the association of long-term glucocorticoid levels with MetS using data of 1405 participants (73.4% women) of the Netherlands Study of Depression and Anxiety. Predictors included hair cortisol and cortisone levels. Outcomes were MetS presence, number of MetS components, and individual component (ie, diastolic blood pressure, waist circumference, and fasting glucose, high-density lipoprotein cholesterol, and triglycerides). We investigated potential interactions with age, sex, and mental health status.

**Results:**

Hair glucocorticoid levels were positively associated with MetS presence (OR = 1.27; 95% CI = 1.11-1.44, and OR = 1.32; 95% CI = 1.14-1.52 for hair cortisol and cortisone, respectively), number of MetS components, waist circumference, and triglyceride levels. Hair cortisol, but not cortisone, was in trend associated with diastolic blood pressure and high-density lipoprotein cholesterol levels. No associations were seen with blood glucose. Of note, the relationship of hair cortisone with MetS was stronger among younger compared to older individuals (OR = 1.95; 95% CI = 1.50-2.54 vs OR = 1.14; 95% CI = .96-1.35 in younger vs older participants).

**Conclusion:**

Long-term biological stress, measured through hair glucocorticoid levels, is associated with MetS presence, especially among younger individuals. Prospective studies need to evaluate the extent to which hair cortisol and cortisone add to standard risk factors when predicting future cardiometabolic diseases.

The metabolic syndrome (MetS) describes a clustering of cardiometabolic complications affecting about 1 of 3 adults in the Western world (1, 2). Importantly, MetS has been identified as a major predictor of cardiovascular disease (CVD), which in turn poses the number 1 global reason of death and disease burden (3). It is therefore essential to understand the complex interplay of mechanisms underlying cardiometabolic deterioration.

A potential contributing factor in the pathogenesis of MetS and CVD is the exposure to stress and stress-related hormones, specifically the glucocorticoid hormone cortisol and its inactive form, cortisone (4). The striking link of Cushing syndrome, a state of chronic extreme glucocorticoid excess, with features of MetS and an increased CVD risk, has long suggested long-term glucocorticoid excess as a catalyst of cardiometabolic deterioration (5). However, it long remained unclear whether the results from such extreme glucocorticoid overabundance can be translated into the relevance of more subtle variations in long-term glucocorticoid levels (eg, from chronic stress).

Thanks to recent advances in scalp hair cortisol and cortisone measurements, researchers can now reliably investigate the association between subtle variations in chronic exposure to stress hormones and cardiovascular health in a quantitative, noninvasive manner. This contrasts with measurements in traditional matrices (saliva, blood, and urine), which reflect short-term glucocorticoid exposure (ranging from minutes up to 24 hours). Importantly, these short-term measures are subject to both diurnal pulsations and day-to-day variations, making them prone to confounding. In contrast, hair glucocorticoid measurements allow for quantifying the average exposure to glucocorticoid levels over months, which reduces the likelihood of confounding due to eg, acute physiological and/or psychological stress (6, 7). Indeed, hair glucocorticoid levels seem more closely associated with cardiometabolic health outcomes, compared to measurements in blood, urine, or saliva (8-10). In a recent meta-analysis, we have demonstrated a strong cross-sectional link between the presence of high hair cortisol levels and the presence of CVD, including data from 5016 individuals (8). Even more recently, we showed hair cortisone to be a significant predictor of incident CVD after controlling for standard risk factors (11). Our results moreover indicated that the association may be stronger among younger compared to older individuals, men compared to women, and, possibly also, among people without mental health problems compared to people suffering from mental health problems.

Despite strong evidence for an association between hair glucocorticoids and CVD, results regarding MetS are less consistent (8). Here, we provide data regarding the relation of hair glucocorticoid levels as a chronic biological stress marker with the presence of MetS in a mixed group of subjects with and without mental health problems. We also tested the potential moderating effects of age, sex, and mental health status on the strength of this association.

## Methods

### Participants

We included subjects from the Netherlands Study of Depression and Anxiety (NESDA), an ongoing longitudinal cohort study on the predictors, course, and consequences of depressive and anxiety disorders. The original NESDA sample consisted of 2981 participants aged 18 to 65 years, including persons with no depressive or anxiety disorder, persons who have had a disorder in the past, and persons with a current depressive and/or anxiety disorder (12). The research protocol was approved by the Ethical Committee of the participating research centers, and all participants provided written informed consent. Hair glucocorticoid levels have been determined in wave 5 of NESDA (6-year follow-up from baseline) to examine their correlation with symptoms of depression and anxiety (13). Of the N = 2256 people who participated in wave 5, hair samples were available for 1604 subjects. Using these data, our research group has previously demonstrated that hair cortisol and cortisone levels can be used to predict future increases in weight and abdominal fat accumulation, known risk factors for the development of MetS and CVD, at 3-year-follow-up (14). For the present analyses, we included participants of whom (1) written consent was available for the use of the hair glucocorticoid data (n = 1559) as well as data regarding (2) the presence/absence of MetS (n = 1462), and who furthermore (3) were not pregnant (n = 1442) and (4) did not use any systemic corticosteroids in the past 3 months (n = 1405). The flowchart of inclusion can be found in Supplementary Fig. S1 (15).

### Hair Glucocorticoid Measurements

Trained research staff collected hair samples from participants who consented and had sufficient hair. Specifically, hair was cut as close to the scalp as possible at the posterior vertex (6). Hair cortisol and hair cortisone were determined in the first 3 cm, using a validated liquid chromatography-tandem mass spectrometry method (LC-MS/MS). The details regarding the extraction and analysis of cortisol and cortisone using this liquid LC-MS/MS have extensively been described previously (16). The inter-assay variation over a 9-month period for cortisol and cortisone was <16%, and the intra-assay variation for cortisol and cortisone was <2.9% and <4.8%, respectively (16).

### MetS Components

Levels of high-density lipoprotein (HDL) cholesterol, glucose, and triglycerides were determined from fasting, morning blood samples using standardized routine laboratory methods (12, 17). Diastolic blood pressure was assessed via an OMRON M4 IntelliSense digital blood pressure monitor, as an average of 2 right-arm measurements. Waist circumference was assessed using a measuring tape at the central point between the highest point of the pelvis and the lowest front rib over light clothing.

Presence of MetS was defined as 3 or more of the following according to the widely used adjusted ATP-III guideline: (1) waist circumference >88 cm in women, >102 cm in men (abdominal obesity); (2) HDL cholesterol <1.03 mmol/L in men, <1.30 mmol/L in women or medication for reduced HDL cholesterol (low HDL cholesterol); (3) triglycerides ≥1.7 mmol/L or medication for hypertriglyceridemia (hypertriglyceridemia); (4) blood pressure ≥130/85 mm Hg or use of antihypertensive medication (hypertension); and (5) fasting plasma glucose ≥5.6 mmol/L or use of antidiabetic medication (hyperglycemia) (18).

### Other Covariates

Other relevant assessed covariates included (1) demographics (sex, age, and educational level [years of education], assessed via self-report) as well as (2) lifestyle factors (smoking [dichotomized into current smoker, nonsmoker at the time of the interview] and alcohol consumption [self-reported average number of drinks per day]), (3) use of medications (including tricyclic antidepressants or medications for low HDL cholesterol, hypertriglyceridemia, hypertension or type 2 diabetes, assessed by inspection of drug containers brought in), (4) hair washing frequency (self-reported on a scale from “1” for < once a week to “4” > 4 times a week), and finally (5) physical activity (assessed via the International Physical Activity Questionnaire) (19). Additionally, we assessed mental health status, defined as the presence of major depression and/or anxiety disorder in the past 6 months according to the Composite International Diagnostic Interview, which classifies diagnoses according to the Diagnostic and Statistical Manual of Mental Disorders-IV criteria (20).

### Statistical Analysis

Baseline characteristics are presented as the mean (SD) or median (interquartile range; p25, p75) in Table 1 by sex. To test potential sex differences, we used chi-squared tests, independent *t*-tests or Mann-Whitney *U* tests, as appropriately depending on the nature (categorical vs continuous) and distribution (normal vs nonnormal) of the data. Before analysis, the variables of hair cortisol, hair cortisone, triglycerides, and blood glucose levels were log10-transformed due to their positively skewed distributions. Log-transformed hair cortisol and cortisone were moreover standardized into *z*-scores for each individual using the following formula: $Z_{Log10\;hair\;cortisol\;or\;cortisone}=\frac{mean-individual\;value}{standard\;deviation}$ to allow the comparison of effect sizes between hair cortisol and hair cortisone. We also *z*-standardized fasting blood glucose, triglycerides, HDL cholesterol levels, diastolic blood pressure, and waist circumference.

**Table 1.** Baseline characteristics of the study sample, stratified by sex

	Level	Female mean (SD) / median (IQR)	Male mean (SD) /median (IQR)		Overall mean (SD) /median (IQR)	Missing (%)
N		1034	371	*P*	1405	
Body mass index		24.80 [22.23, 28.73]	25.59 [22.97,28.73]	.051	25.01 [22.40, 28.73]	0
Age, y		49.00 [37.00, 59.00]	50.00[34.50,60.00]	.413	49.00 [36.00, 59.00]	0
Educational level (years of education)		12.93 (3.34)	12.90 (3.27)	.867	12.92 (3.32)	0
Physical activity (IPAQ category (%))	Low	180 (17.4)	95(25.6)	.001	275 (19.6)	0
	Medium	464 (44.9)	134 (36.1)		598 (42.6)	
	High	390 (37.7)	142 (38.3)		532 (37.9)	
Current smoker (%)		263 (25.4)	116 (31.3)	.035	379 (27.0)	0
Average number of alcoholic drinks per day		0.79 (1.76)	2.31 (3.53)	<.001	1.20 (2.45)	0
Presence of a mental disorder past 6 months (depression and/or anxiety, %)		305 (29.5)	86 (23.2)	.024	391 (27.8)	0
Hair cortisol (pg/mg)		3.23 [2.06,5.66]	3.40 [2.28,6.24]	.054	3.27 [2.12, 5.76]	2.7
Hair cortisone (pg/mg)		10.10 [6.95,14.79]	12.53 [9.09,17.37]	<.001	10.63 [7.55, 15.29]	.1
Hair washing frequency category (%)	1	54 (5.2)	27 (7.3)	<.001	81 (5.8)	0
	2	285 (27.6)	68 (18.3)		353 (25.1)	
	3	386 (37.3)	103 (27.8)		489 (34.8)	
	4	309 (29.9)	173 (46.6)		482 (34.3)	
Presence of the metabolic syndrome (%)		240 (23.2)	138 (37.2)	<.001	378 (26.9)	0
Number of metabolic syndrome components*^a^* (%)	0	293 (28.3)	35 (9.4)	<.001	328 (23.3)	0
	1	296 (28.6)	105 (28.3)		401 (28.5)	
	2	205 (19.8)	93 (25.1)		298 (21.2)	
	3	141 (13.6)	81 (21.8)		222 (15.8)	
	4	60 (5.8)	39 (10.5)		99 (7.0)	
	5	39 (3.8)	18 (4.9)		57 (4.1)	
Triglyceride levels (mmol/L)		1.00 [0.71, 1.40]	1.30 [0.98, 1.91]	<.001	1.08 [0.77, 1.54]	0.1
HDL cholesterol (mmol/L)		1.67 (0.43)	1.32 (0.35)	<.001	1.58 (0.44)	0.2
Diastolic blood pressure (mm Hg)		77.23 (9.88)	81.43 (10.01)	<.001	78.34 (10.08)	0.3
Blood glucose levels (mmol/)		5.25 [4.90, 5.60]	5.50 [5.20, 6.00]	<.001	5.30 [5.00, 5.70]	1.2
Waist circumference (cm)		87.00 [78.25, 97.00]	96.00 [89.00, 105.00]	<.001	90.00 [81.00, 100.00]	0
Use of fibrates (%)		0 (0.0)	2 (0.5)	.119	2 (0.1)	0
Use of antihypertensive medication (%)		187 (18.1)	89 (24.0)	.017	276 (19.6)	0
Use of antidiabetic medication (%)		30 (2.9)	20 (5.4)	.040	50 (3.6)	0
Use of tricyclic antidepressants (%)		40 (3.9)	9 (2.4)	.257	49 (3.5)	0

Abbreviations: IPAQ, International Physical Activity Questionnaire; IQR, interquartile range.

^*a*^Adjusted ATP-III criteria. Results for physical activity and hair washing frequency were imputed.

The primary analyses included logistic regressions with hair glucocorticoid levels (hair cortisol and cortisone) as predictors and the presence/absence of MetS as the outcome, both crude (model 1) and adjusted for age, sex, educational level, smoking, alcohol consumption, physical activity, mental health status, use of tricyclic antidepressants, and hair washing frequency (model 2). Interaction terms were added to model 2 to test for the presence/absence of group differences regarding age, sex, and mental health. To improve interpretability, age (in years) was standardized before adding it to the models using the same formula as described previously. Additionally, stratified logistic regressions were performed according to age (≤49 years vs ≥ 50 years, median split), sex (men vs women), and mental health status (no current mental disorder vs presence of depression and/or anxiety disorder in the past 6 months).

Secondary analyses included linear regressions using hair glucocorticoid levels (hair cortisol and cortisone) as predictors and components of MetS: number of fulfilled MetS components, HDL-cholesterol, glucose, triglycerides, blood pressure, and waist circumference. Again, analyses were performed crude (model 1) and adjusted for age, sex, educational level, smoking, alcohol consumption, physical activity, mental health status, use of tricyclic antidepressants, and hair washing frequency. Additionally, we also adjusted those models for use of relevant medications (ie, antidiabetic medication use was added to the model predicting blood glucose levels; use of fibrates was added to the models predicting triglycerides and HDL cholesterol levels; and antihypertensive medication use was added to the model predicting blood pressure) (model 2).

To overcome possible selection bias because of missing data (21, 22), we applied multiple imputation regarding missing values of physical activity and hair washing frequency under the assumption that they were missing at random (7%; 0.1% missing respectively). Specifically, we applied predictive mean matching using the mice package in R (23). All other variables included in the models either (1) did not include any missing values (demographics and lifestyle factors) or (2) posed either predictor or outcome of the models, precluding imputation (see Table 1 for baseline characteristics and % of missing values). All characteristics were used as predictors to impute missing values of physical activity and hair washing frequency. The results of regression analyses reflect the pooled results of 5 iterations. A sensitivity analysis was performed on subjects with complete data only (see Supplementary Material S2 (15)). Model fit was compared between models using the likelihood-ratio test. Model diagnostics, including the likelihood-ratio test, were performed using the first imputed dataset.

Logistic regression analyses were corrected for multiple testing by setting significance at 0.05/8 = 0.00625 (accounting for the main analysis plus testing of three interactions of the 2 predictors hair cortisol and cortisone, thus 8 tests in total). For linear regression analyses, significance was set at 0.05/2 = 0.025 to account for testing all models regarding 2 different predictors. All statistical analyses were performed in R 4.3.2 (23).

## Results

### Baseline Characteristics

A total of N = 1405 participants were included in the final analysis (N = 1034 women [73.6%]; see Supplementary Fig. S1 (15) for the inclusion flowchart). Because of technical difficulties regarding laboratory analyses, hair cortisol was not measurable in n = 38 of these participants (2.7%) and hair cortisone was not measurable in 1 participant (0.1%). Overall, men had unhealthier lifestyle behaviors (less physical activity, more frequent smoking, and more alcohol consumption, all *P* < .05), and had less favorable metabolic health outcomes across all MetS components compared to women (all *P* < .001). Men also reported having suffered from depression and/or anxiety disorder in the recent past less frequently compared to women, and they more often took antihypertensives and/or antidiabetic medication (*P* < .05). For details, see Table 1.

### Hair Cortisol and Cortisone Levels in Relation to Components of MetS

As expected, hair cortisol and cortisone strongly correlated with each other (Spearman rho = 0.624, *P* < .001). In both crude and adjusted analyses (model 1 and model 2, respectively), hair cortisol and hair cortisone were strongly associated with the presence of MetS (all *P* < .001, see Table 2 for coefficients of hair glucocorticoids, see Supplementary Table S3 (15) for all coefficients).

**Table 2.** Logistic regressions regarding associations of hair cortisol and cortisone with MetS

	Model 1 (univariate)						Model 2 (adjusted)					
	Odds ratio	95% CI (lower)	95% CI (upper)	*P*-value	N (observations)	N (cases)	Odds ratio	95% CI (lower)	95% CI (upper)	*P*-value	N (observations)	N (cases)
**Whole sample**												
Hair cortisol*^a^*	1.372	1.220	1.544	<.001	1367	367	1.268	1.113	1.444	<.001	1367	367
Hair cortisone*^a^*	1.540	1.351	1.757	<.001	1404	378	1.317	1.139	1.522	<.001	1404	378

Model 2 was adjusted for age, sex, educational level, smoking, alcohol consumption, physical activity, mental health status, use of tricyclic antidepressants, and hair washing frequency.

Abbreviation: 95% CI, 95% confidence interval.

^*a*^log10 transformed, *z*-score.

**Figure 1.**
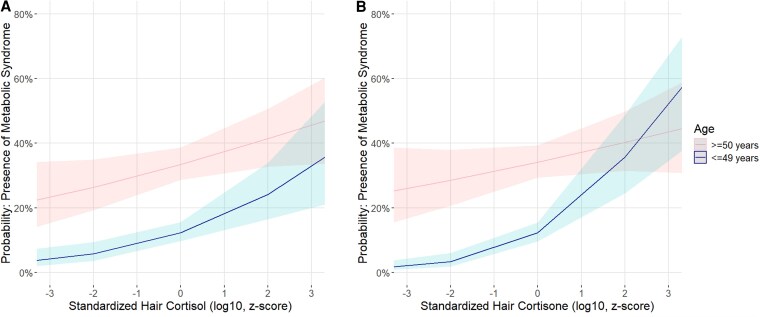
Logistic regressions regarding associations of hair cortisol and cortisone with MetS by age group. Shows the predicted probability of having the MetS, according to baseline hair glucocorticoid levels (pg/mg, log10-transformed, and standardized into *z*-scores), split by age category (≥50 years or ≤49 years, median split). Figure 1A refers to the predicted probability of having MetS according to hair cortisol; Fig. 1B refers to the predicted probability of having MetS according to hair cortisone. Analyses were adjusted for age, sex, educational level, smoking, alcohol consumption, physical activity, mental health status, use of tricyclic antidepressants, and hair washing frequency. The red line indicates the predicted probability, with red bands indicating 95% CI for older individuals. The blue line indicates predicted probability for the younger individuals, with blue bands indicating 95% CI. The graphical representation is based on the first imputed dataset after multiple imputation of the covariate physical activity.

Adding an interaction term hair cortisone * age to model 2 revealed a significant interaction effect of hair cortisone and age in predicting the presence of MetS (OR_hair cortisone*age_ = 0.78; 95% CI, .66–.92, *P* = .003; see Fig. 1), and improved model fit compared to model 2 according to the likelihood-ratio test (*P* = .003). The finding indicates a significantly stronger association between hair cortisone and the presence of MetS among younger participants. The interaction of hair cortisol * age, however, was not statistically significant (OR_hair cortisol*age_ = 0.93; 95% CI, .81-1.08, *P* = .342) and did not contribute to an improved model fit according to the likelihood-ratio test (*P* = .352). Age-stratified analyses revealed larger ORs for both hair cortisol and cortisone among younger compared to older participants (OR = 1.52; 95% CI, 1.22-1.88 vs OR = 1.20; 95% CI, 1.02-1.40 for hair cortisol, and OR = 1.95; 95% CI, 1.50-2.54 vs OR = 1.14; 95% CI, .96-1.35 for hair cortisone in model 2, see Supplementary Table S4 (15)).

Interaction terms of hair cortisol or cortisone with mental health status did not show significant interaction effects (OR_hair cortisol*mental health_ = 0.97; 95% CI, .72-1.30, *P* = .839 and OR_hair cortisone*mental health_ = 1.01; 95% CI, .73-1.39, *P* = .948). Similarly, interaction terms of either hair cortisol or cortisone with sex did not reveal any significant interaction effects (OR_hair cortisol*sex_ = 1.12; 95% CI, .83-1.49, *P* = .460 and OR_hair cortisone*sex_ = 1.01; 95% CI, .72-1.40, *P* = .967). In line with this, the likelihood-ratio test did not indicate improved model fit after adding the interaction terms for mental health status or sex compared to model 2 (all *P* > .400).

### Hair Cortisol and Cortisone Levels in Relation to MetS Components

After adjustment for confounders, both hair cortisol and cortisone closely positively correlated with the number of an individual's fulfilled MetS components (both *P* < .001). When looking at all MetS components separately, hair cortisol and cortisone were significantly positively correlated with waist circumference (all *P* < .001) and triglyceride levels (all *P* < .025 after adjustment for confounders). Additionally, hair cortisol was in trend associated with diastolic blood pressure and HDL cholesterol levels, but this was not found for hair cortisone after adjustment (*P* = .034 and *P* = .035, respectively, for hair cortisol; *P* = .806 and .087, respectively, for cortisone). Only for blood glucose, no significant associations were found with either hair cortisol or cortisone levels after adjustment. For details, see Table 3.

**Table 3.** Linear regressions regarding associations of hair cortisol and cortisone with MetS components

	Model 1 (univariate)					Model 2 (adjusted)				
	β	95% CI (lower)	95% CI (upper)	*P*-value	N	β	95% CI (lower)	95% CI (upper)	*P*-value	N
**Number of MetS components**										
Hair cortisol*^a^*	.235	.163	.308	<.001	1367	.142	.076	.207	<.001	1367
Hair cortisone*^a^*	.271	.200	.342	<.001	1404	.136	.070	.202	<.001	1404
**Waist circumference (cm)** *^b^*										
Hair cortisol*^a^*	.148	.095	.200	<.001	1367	.092	.045	.140	<.001	1367
Hair cortisone*^a^*	.185	.134	.237	<.001	1404	.094	.045	.142	<.001	1404
**Triglyceride levels (log10)** *^b^*										
Hair cortisol*^a^*	.127	.074	.180	<.001	1365	.063	.014	.113	.013	1365
Hair cortisone*^a^*	.164	.112	.215	<.001	1402	.069	.019	.119	.007	1402
**Blood glucose levels (log10)** *^b^*										
Hair cortisol*^a^*	.127	.074	.180	<.001	1350	.029	−.015	.074	.197	1350
Hair cortisone*^a^*	.153	.101	.205	<.001	1387	.028	−.017	.073	.225	1387
**Diastolic blood pressure (mm Hg)** *^b^*										
Hair cortisol*^a^*	.131	.078	.184	<.001	1363	.054	.004	.104	.034	1363
Hair cortisone*^a^*	.116	.063	.168	<.001	1400	.006	−.044	.057	.806	1400
**HDL cholesterol (mmol/L)** *^b^*										
Hair cortisol*^a^*	−.050	−.103	.003	.066	1364	−.053	−.102	−.004	.035	1364
Hair cortisone*^a^*	−.071	−.123	−.018	.008	1401	−.043	−.093	.006	.087	1401

Model 2 was adjusted for age, sex, educational level, smoking, alcohol consumption, physical activity, mental health status, use of tricyclic antidepressants, and hair washing frequency. Additionally, models were adjusted for use of relevant medications (ie, antidiabetic medication use was added to the model predicting blood glucose levels, use of fibrates was added to the models predicting triglycerides, and high-density lipoprotein cholesterol levels; and antihypertensive medication use was added to the model predicting blood pressure).

Abbreviations: 95% CI, 95% confidence interval; MetS, metabolic syndrome.

^*a*^log10-transformed and *z*-standardized.

^*b*^ *z*-standardized.

## Discussion

We investigated the cross-sectional association of chronic biological stress, measured as hair glucocorticoid levels, with the presence of MetS and its components (abdominal obesity, hypertriglyceridemia, hyperglycemia, hypertension, low HDL cholesterol levels). We also tested potential moderating effects by age, sex, and mental health status on the strength of this association. In short, we found a positive association between hair glucocorticoid levels and the presence of MetS. For hair cortisone, this was stronger among younger compared to older subjects, which is in line with recent findings regarding cardiovascular disease. Neither sex nor mental health status (healthy vs presence of depression and/or anxiety in the past 6 months) moderated the association. All MetS components, except blood glucose levels, were significantly or in trend associated with levels of hair glucocorticoids, as was the total number of fulfilled MetS components.

To our knowledge, this is the largest study regarding the association of hair glucocorticoid levels with MetS and the first to investigate the role of potential moderators of this relationship. In a recent systematic review and meta-analysis, we showed that current evidence regarding the relation between hair glucocorticoid levels and the presence of MetS is very heterogeneous. Of 8 available articles, only 3 reported the expected positive association; 1 study in patients with HIV even reported a negative association (8). Nevertheless, the findings presented in the current study are largely in line with the largest previous study (N = 1258) regarding hair glucocorticoid levels in relation to MetS (24). Similar to our findings, the authors reported a strong positive association between hair glucocorticoid levels and the presence of MetS, despite small differences regarding the different MetS components. The concordance of our results is interesting in view of notable differences regarding the composition of the 2 study populations: while the sample of the previous study was predominantly composed of men (84.8%), ours was predominantly female (73.6%). Moreover, a substantial part of our study population was suffering from depression and/or anxiety disorder in the recent past (27.8%), which is likely higher than in the previous study. We note that the prevalence of depression and anxiety is not described in the previous article (24). However, a prevalence of 27.8% is substantially higher than the overall prevalence of depression and anxiety in comparable populations (25, 26). Together with the findings of our subgroup and interaction analyses, this concordance of study results points toward the possibility that the association between hair glucocorticoid levels and cardiometabolic health is largely independent of sex and mental health status.

We also investigated the link between hair glucocorticoid levels and the different MetS components to gain a better understanding of what drives the observed association. Glucocorticoid levels were associated with all components except glucose levels. Together, the findings indicate that the relation between hair glucocorticoid levels and MetS is primarily driven by associations with blood pressure, triglycerides, low HDL cholesterol levels, and abdominal fat accumulation, rather than blood glucose levels. Importantly, of all MetS components, waist circumference was most strongly related to hair glucocorticoid levels in our analysis, suggesting that a large part of the association between long-term glucocorticoid excess and cardiometabolic deterioration is mediated by abdominal fat accumulation. Indeed, there is a strong link between long-term hypercortisolism and adiposity. For instance, long-term hypercortisolism has been shown to increase hedonic eating tendencies via actions on hypothalamic and peripheral satiety signaling, which predispose to overconsumption and, consequently, weight gain (27-29). Furthermore, glucocorticoids also exert direct effects on adipocytes through local glucocorticoid receptor signaling. For instance, while glucocorticoids induce acute increases in lipolysis in adipocytes in the subcutaneous depot, in the visceral depot they exert relatively more lipogenic effects, culminating in more visceral fat accumulation (30, 31).

Notably, hair cortisone was more strongly associated with the presence of MetS and number of fulfilled MetS components than hair cortisol. This is in line with previous data regarding obesity measures (9), MetS (24), and CVD (11), suggesting that hair cortisone might be a better marker for cardiometabolic risk than the active form cortisol. Mechanistically, cortisone has been hypothesized to represent a more stable marker for the long-term systemic reservoir of available glucocorticoids in the body, as evidence suggests that a specific fraction of systemic cortisol levels may be converted into the inactive form cortisone at the scalp before being stored in hair (24, 32). This might also explain why levels of hair cortisone are consistently reported to be higher than levels of hair cortisol. Additionally, laboratory measurements of hair cortisone are usually easier and more reliable than those of hair cortisol due to smaller measurement errors (33). Altogether, measuring hair cortisone in addition to hair cortisol provides useful additive insights regarding an individual's long-term stress exposure and is therefore likely more useful than measuring hair cortisol alone. However, hair cortisol still remains by far the more frequently measured biomarker in the context of long-term glucocorticoid exposure in relation to cardiometabolic health (8).

Interestingly, our results indicate that the association of hair glucocorticoid levels, particularly hair cortisone, with MetS is stronger among younger compared to older people. This is in line with findings regarding CVD (8, 11), and with studies reporting that the relative risk contributions of traditional cardiovascular risk factors decline with increasing age (due to an increased number of “competing” health risks) (34, 35). Together, the results indicate that other pathways have a larger contribution to cardiovascular events among the elderly. Moreover, as hair glucocorticoid levels notably increase with age (36), it is tempting to speculate that higher hair cortisol and cortisone levels in younger individuals may reflect accelerated biological aging, as these individuals resemble older individuals with regard to cardiometabolic risk. In addition, evidence indicates that glucocorticoid sensitivity decreases with age (37-39), which would render younger individuals more susceptible to the effects of long-term glucocorticoid excess. Future studies should further investigate the effects of age on the relation between long-term glucocorticoid exposure and cardiometabolic health. Moreover, although our analysis did not reveal moderating effects of sex or mental health status regarding the association between hair glucocorticoids and cardiometabolic health, this does not preclude potential subgroup effects (eg, according to menstrual status [menopause vs nonmenopause] or type of depression/anxiety [eg, typical vs atypical]). This may be especially relevant in view of different depression subtypes relating differently to weight (40-43). Previous results have not shown a consistent link between mental health (eg, depression/anxiety) and hair glucocorticoid levels (36, 44), contrasting findings regarding anthropometrics in association with hair glucocorticoids (9). In view of the variability and complexity of depressive and anxiety symptomatology, it is possible that specific disease subgroups may differentially be linked to hair glucocorticoid levels via differential associations with HPA axis activation, as is also suggested by previous data (45). Although this presents a very interesting field for future research, analyses of, for example, depression subtypes were beyond the scope of the present analysis. Future studies should expand our current analysis toward deeper investigation in the context of mental health and sex.

Limitations of our study include that, inherent to the population of the Netherlands, the NESDA cohort is mainly composed of Caucasian participants with >97% of the original cohort being of Dutch nationality and >94% having Northern European ancestry. This may hamper generalizability to other populations and ethnic minorities living in Western countries such as the Netherlands. This may be relevant in view of evidence suggesting potential ethnic differences in glucocorticoid sensitivity and metabolism, which may render other ethnicities more prone to adverse (metabolic) effects of glucocorticoid excess, which is an interesting subject for further research (46-50). Also, we did not have data regarding participants’ diets and can therefore not exclude potential effects of nutrition or nutritional status on our analysis. We note that, so far, there is little evidence regarding the association of diet and glucocorticoid metabolism or glucocorticoid storage in hair (51, 52). Strikingly, obesity (reflecting a state of long-term overnutrition), is associated with increased hair cortisol and cortisone levels (9), whereas anorexia nervosa (reflecting long-term malnutrition), is not associated with altered levels of hair glucocorticoids, compared to healthy-weight controls, precluding effects of nutritional status on glucocorticoid storage in hair per se (53). However, anorexia nervosa itself is associated with hair loss, presumably as a consequence of nutrient deficiency (54, 55). Next, we note here that while hair glucocorticoid levels are closely linked to physical stressors such as eg, shift work/sleep deprivation or diseases such as Cushing syndrome (7 , 56, 57), their relation with mild autonomous cortisol secretion and mild variations in self-reported perceived stress is inconsistent (36, 44, 58). Thus, long-term biological stress (reflected in hair glucocorticoid levels) and psychological stress (reflected eg, in questionnaires) likely reflect different aspects of the stress response. In future studies, it would be very valuable to include both. Finally, due to the cross-sectional design of this study, we cannot draw conclusions on causality of the observed association. Although ample evidence suggests that overexposure to glucocorticoids often precedes weight gain (14) and cardiometabolic deterioration (5, 11, 29), it is also possible that suffering from the metabolic syndrome can function as a biological stressor and increase HPA activity. The idea of a bidirectional association of glucocorticoid excess and metabolic deterioration is suggested by findings in other (short-term) cortisol matrices in response to weight loss (59-61). However, to our knowledge, this has not yet been investigated with regard to hair glucocorticoid levels. Future studies are needed to investigate the extent to which hair glucocorticoid levels, reflecting subtle variations in long-term biological stress, can be used to predict an individual's risk of developing MetS. Another very interesting field of future studies includes the investigation of potential mediators of the relation between hair glucocorticoid levels and cardiometabolic health, such as markers of biological aging.

In summary, we demonstrated a positive cross-sectional link between long-term biological stress, measured as hair glucocorticoid levels, and the presence of MetS and its components, which was particularly strong in younger adults with regard to hair cortisone. In the future, these findings can be used to identify individuals with increased cardiovascular risk.

## Data Availability

The data that support the findings of this study are available on request from the NESDA cohort study. The data sharing policy of NESDA and information on how to request for access to study data is available at https://www.nesda.nl/researchers/about-nesda/.

## Abbreviations

CVD: cardiovascular disease
HDL: high-density lipoprotein
LC-MS/MS: liquid chromatography tandem mass spectrometry
MetS: metabolic syndrome
NESDA: Netherlands Study of Depression and Anxiety
